# Diagnosing STEMI in right then left bundle branch block pattern ventricular escape rhythm (case report)

**DOI:** 10.1093/omcr/omac126

**Published:** 2022-11-24

**Authors:** Mochamad Yusuf Alsagaff, Muhammad Dedy Pratama, I G N Iswan Rahmadi Ranuh, Terrence Timothy Evan Lusida

**Affiliations:** Department of Cardiology and Vascular Medicine, Faculty of Medicine, Airlangga University-Dr Soetomo General Hospital Surabaya, Surabaya, Indonesia; Cardiology Department, Universitas Airlangga Hospital, Surabaya, Indonesia; Department of Cardiology and Vascular Medicine, Faculty of Medicine, Airlangga University-Dr Soetomo General Hospital Surabaya, Surabaya, Indonesia; Department of Cardiology and Vascular Medicine, Faculty of Medicine, Airlangga University-Dr Soetomo General Hospital Surabaya, Surabaya, Indonesia; Department of Cardiology and Vascular Medicine, Faculty of Medicine, Airlangga University-Dr Soetomo General Hospital Surabaya, Surabaya, Indonesia

## Abstract

Electrocardiography is the fastest bedside tool for rapidly identifying patients with acute coronary syndromes who require emergency reperfusion therapy. Some of the circumstances that make identification more complex are bundle branch block patterns. ST elevation in the right bundle branch block (RBBB) can still be detected, but the left bundle branch block (LBBB) must use specific criteria such as Sgarbossa and Barcelona. We present a patient with anteroseptal ST-segment elevation (STEMI), total AV block (TAVB) with ventricular escape rhythm RBBB pattern, and then turned into a LBBB pattern. Fortunately, it immediately turned into sinus rhythm after reperfusion therapy. It is essential to be able to identify STEMI in patients with BBB patterns. In addition, to provide the best possible outcomes for the patient, we must understand that the best way to manage STEMI with TAVB is to immediately install a temporary pacemaker and initiate reperfusion therapy.

## INTRODUCTION

Accurate and rapid identification of acute coronary occlusion is critical for determining emergency reperfusion therapy [1]. Electrocardiography (ECG) is the fastest bedside tool to help identify it, but ECG is not without its limitations. Some conditions that make identification more complex are bundle branch block patterns such as left or right bundle branch block (LBBB/RBBB) or ventricular escape rhythm. ST elevation in RBBB can still be detected, but LBBB must use specific criteria such as Sgarbossa and Barcelona. These criteria are essential because an ECG of a patient with LBBB can show changes in the ST segment (elevation or depression) even though the patient does not have ACS [2]. In this case, we report exciting points of ECG learning. The patient experienced acute anteroseptal STEMI, total AV block (TAVB) with escape ventricular rhythm RBBB morphology, then LBBB morphology, and returned to sinus rhythm immediately after reperfusion.

## CASE REPORT

**Figure 1:**
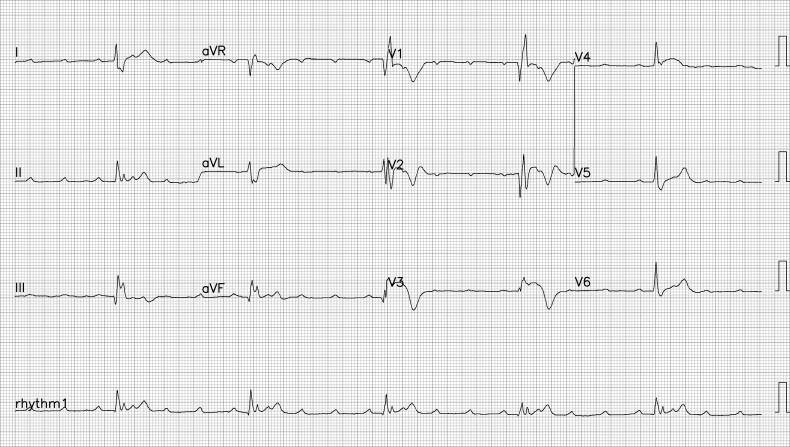
The patient’s ECG at the previous hospital showed total AV block with RBBB pattern escape rhythm, and ST-segment elevation in anteroseptal leads (V1–V4).

**Figure 2:**
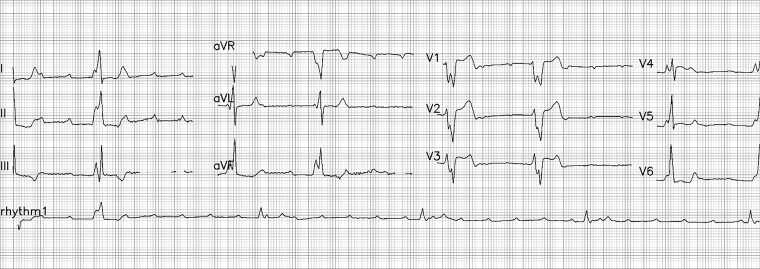
The patient’s post-thrombolytic ECG showed total AV block with LBBB pattern escape rhythm.

**Figure 3:**
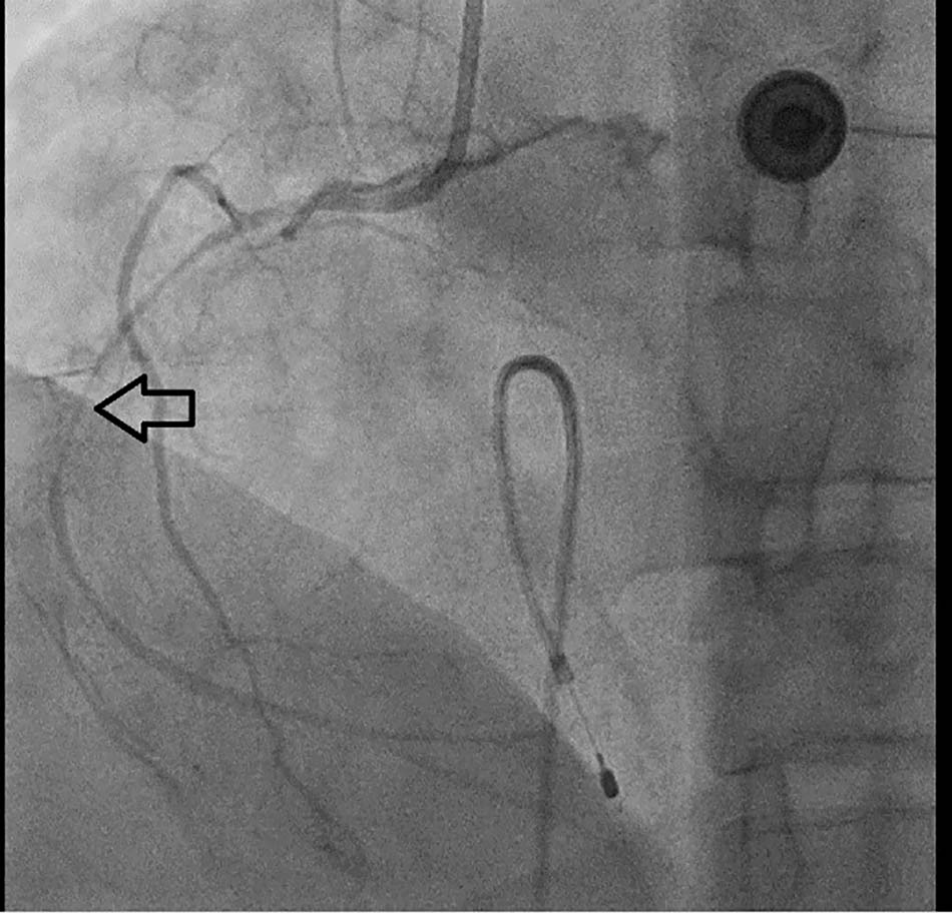
Diagnostic coronary angiography (DCA) showed that the right coronary arteries (RCAs) had critical stenosis 99% in the middle segment.

**Figure 4:**
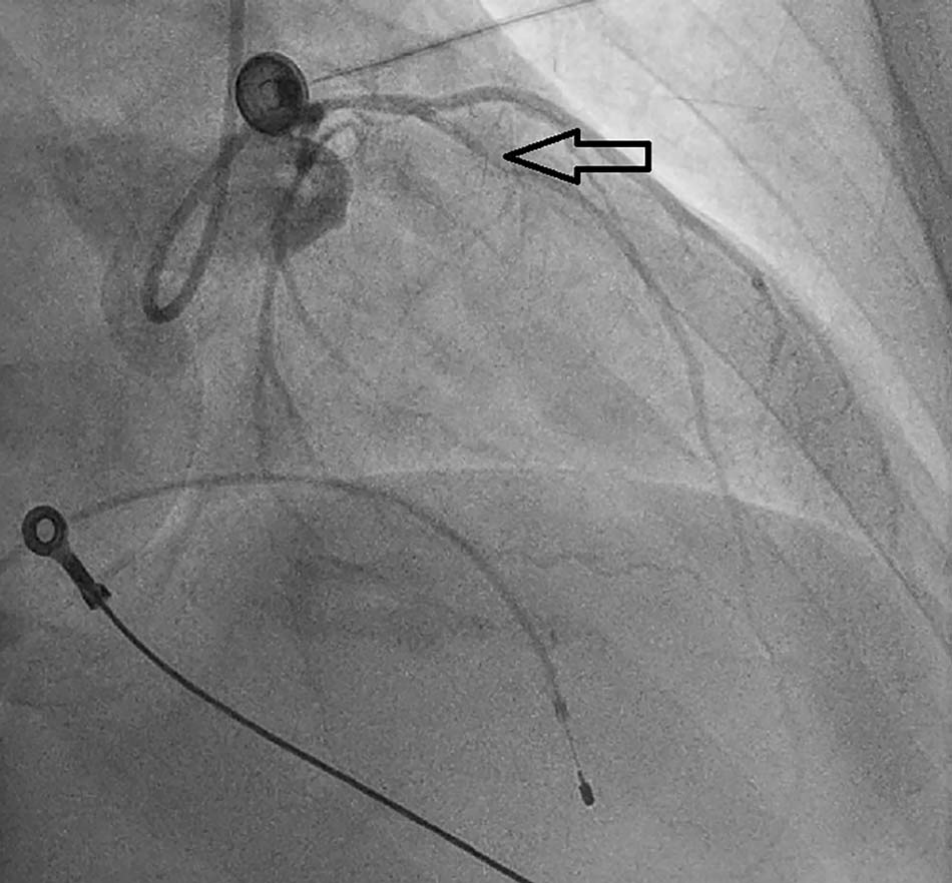
DCA showed LAD with critical stenosis of 99% in the middle segment and significant stenosis of 80% in the distal segment.

**Figure 5:**
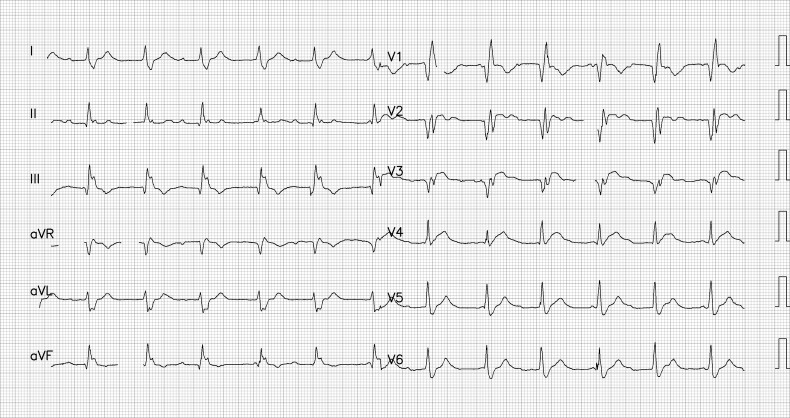
Patient’s ECG after successfully performing complete revascularization procedure and installing a temporary pacemaker. The ECG with standby off pacemaker showed total AV block with RBBB pattern escape rhythm.

**Figure 6:**
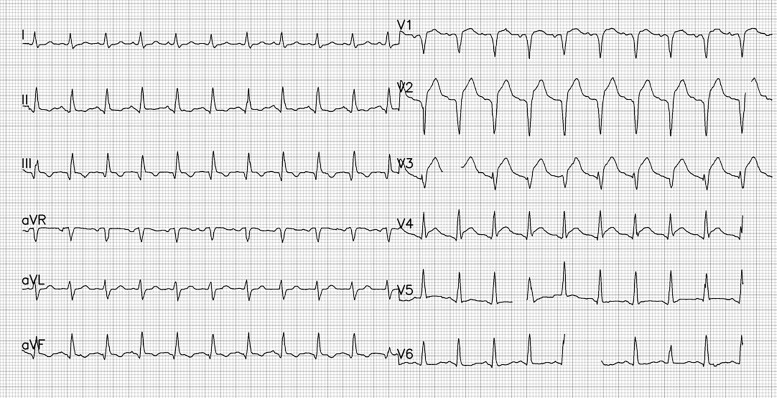
Patient’s ECG 24 hours after revascularization. It showed sinus rhythm and normal axis without any intraventricular block.

A man in his late 40s was referred to our emergency department (ED) for reperfusion. Six hours prior to his previous hospitalization, he had sudden, persistent chest pain (pain rating of 5/10), diaphoresis, nausea, vomiting and fatigue. Total AV block with RBBB pattern escape rhythm and ST-segment elevation in the anteroseptal leads (V1–V4) were observed on an ECG from the previous hospital (Fig. 1). Due to his unstable hemodynamic and severe bradycardia, he was given a 5 μg/min epinephrine infusion. At our ED, he was still experiencing chest pain (pain scale 4/10) with an onset time of 10 hours, stable hemodynamics with vasoactive support and the same ECG pattern. While waiting for an RT-PCR COVID-19 swab test (pandemic era), a percutaneous attachment temporary pacemaker (TPM) and thrombolytic strategy were implemented. A post-thrombolytic ECG revealed a total AV block with LBBB pattern escape rhythm (Fig. 2). Alterations in hemodynamics, such as a decrease in hemodynamic status followed by a sign of shock, coincide with the ECG changes. As soon as negative COVID-19 screening result was obtained, the patient was immediately transferred to the Cath Lab for rescue PCI and transvenous pacemaker placement. The coronary angiography revealed that the right coronary arteries (RCAs) and left anterior descendent (LAD) had critical stenosis (99%) in the middle (Fig. 3), and the LAD had significant stenosis (80%) in the distal (Fig. 4). A cardiac arrest episode occurred during RCA’s angiography, and we immediately did cardiopulmonary resuscitation for less than 3 minutes. After the patient had regained spontaneous circulation, we decided to finish the reperfusion by opening the RCA and LAD segment. Each sirolimus eluting stent (SES) was successfully placed in the osteal-mid RCA with two overlapping SES, and the proximal-mid LAD with two overlapping SES in order to achieve TIMI Flow III. After the procedure, the ECG with the pacemaker on standby showed a return of the TAVB with RBBB pattern (Fig. 5). After 24 hours post revascularization, the patient’s ECG changed significantly to sinus rhythm and normal axis without any intraventricular block (Fig. 6). We removed the TPM on the second day of hospitalization. He was discharged after the seventh day of hospitalization.

## DISCUSSION

Unlike inferior MI, the pathogenesis and prognosis of TAVB in anteroseptal or anterior MI are distinct. Ninety percent of patients with nodal, supra-Hisian or lesions above the bundle of His have occlusion of the RCA segment or inferior myocardial wall. The escape rhythm manifested in these lesions exhibits a narrow QRS complex, either reversible or transient, and the patient is expected to recover within a week. Patients with this ailment typically have asymptomatic bradycardia (40–60 beats per minute) and low mortality risk [3]. In comparison, intranodal or infra-Hisian lesions (distal to the AV node) are associated with occlusion in the LAD segment or anterior wall of the myocardium. This lesion manifests an escape rhythm characterized by a wide QRS complex and occurs within 24 hours. The development of a new BBB at this lesion suggests a significant anterior infarction with an extremely high mortality rate (80%) [4]. In addition, TAVB due to acute LAD occlusion will provide BBB/hemiblock pattern to describe the extent of MI. As in our case, the ventricular escape rhythm that appears had a RBBB pattern initially. Although the existence of RBBB may confound the diagnosis of STEMI, it is not too difficult to diagnose. Immediately after thrombolysis, the ECG changed to LBBB ventricular escape rhythm pattern. Interestingly, additional ECG observations revealed that it did not meet the diagnostic criteria for STEMI in LBBB. Sgarbossa *et al*. have three criteria for diagnosis of acute MI with LBBB: (1) ST-segment, elevation ≥1 mm in any lead with positive QRS (V4, V5, V6, Lead 1 and AVL); (2) ST-segment depression ≥1 mm in V1, V2, and V3 and (3) ST-segment elevation ≥5 mm in any lead with discordant QRS (V1, V2, V3) [5]. Several case reports have also reported alternating BBB. This may be related to variances in the origin of ventricular impulses and the duration of the conduction branch refractory periods during the R-R interval [6]. Treatment for patients with electrical instability due to bundle branch block is TPM placement, and for patients with cardiogenic shock due to coronary artery occlusion is reperfusion. These two procedures are the cornerstone of treatment for patients diagnosed with STEMI and BBB. After undergoing these two main procedures, the patient’s clinical and hemodynamic status continued to improve. On the second day of the patient’s hospitalization, there was an improvement in heart rhythm to sinus rhythm with narrow QRS and thus no longer a BBB pattern. However, ST-segment elevation was still seen in leads V1-V5 of the patient’s ECG. As the patient’s hemodynamics improved, vasoactive support was no longer needed. Furthermore, the patient was discharged from the hospital on the seventh day of the hospitalization with oral therapy including high-intensity statins and dual antiplatelet drugs. The patient planned a monthly follow-up at the outpatient clinic for further monitoring.
